# Production of recombinant protein by a novel oxygen-induced system in *Escherichia coli*

**DOI:** 10.1186/1475-2859-13-50

**Published:** 2014-04-07

**Authors:** Antonino Baez, Nadim Majdalani, Joseph Shiloach

**Affiliations:** 1 Biotechnology Core Laboratory, National Institute of Diabetes and Digestive and Kidney Diseases, National Institutes of Health, Bethesda, MD 20892, USA; 2 National Cancer Institute, National Institutes of Health, Bethesda, MD 20892, USA

**Keywords:** s*oxS* promoter, Protein oxidation, GFP oxidation, High cell density, Carbonyl groups

## Abstract

**Background:**

The SoxRS regulon of *E. coli* is activated in response to elevated dissolved oxygen concentration likely to protect the bacteria from possible oxygen damage. The *soxS* expression can be increased up to 16 fold, making it a possible candidate for recombinant protein expression. Compared with the existing induction approaches, oxygen induction is advantageous because it does not involve addition or depletion of growth factors or nutrients, addition of chemical inducers or temperature changes that can affect growth and metabolism of the producing bacteria. It also does not affect the composition of the growth medium simplifying the recovery and purification processes.

**Results:**

The *soxS* promoter was cloned into the commercial pGFPmut3.1 plasmid creating pAB49, an expression vector that can be induced by increasing oxygen concentration. The efficiency and the regulatory properties of the *soxS* promoter were characterized by measuring the GFP expression when the culture dissolved oxygen concentration was increased from 30% to 300% air saturation. The expression level of recombinant GFP was proportional to the oxygen concentration, demonstrating that pAB49 is a controllable expression vector. A possible harmful effect of elevated oxygen concentration on the recombinant product was found to be negligible by determining the protein-carbonyl content and its specific fluorescence.

By performing high density growth in modified LB medium, the cells were induced by increasing the oxygen concentration. After 3 hours at 300% air saturation, GFP fluorescence reached 109000 FU (494 mg of GFP/L), representing 3.4% of total protein, and the cell concentration reached 29.1 g/L (DW).

**Conclusions:**

Induction of recombinant protein expression by increasing the dissolved oxygen concentration was found to be a simple and efficient alternative expression strategy that excludes the use of chemical, nutrient or thermal inducers that have a potential negative effect on cell growth or the product recovery.

## Background

It has been established that in response to high dissolved oxygen of 300% air saturation, *E. coli* activates the SoxRS regulon which activates the transcription of the *soxS* and the *sodA* genes [1]. The *sodA* gene encodes the manganese-superoxide dismutase (SOD), which reduces the oxidative stress caused by molecular oxygen, allowing the cells to grow uninterrupted at 300% oxygen [2,3]. Baez et al. [1] showed that in response to high oxygen concentrations, *soxS* expression is increased 16 fold, making it a possible candidate for inducing recombinant protein expression in response to high oxygen concentrations. Expression of recombinant proteins is often achieved by coupling the recombinant protein gene to promoters of genes that are activated in response to the presence or absence of a specific factor. Among the known examples for such couplings are the expression systems of the methylotrophic yeast *Pichia pastoris*, where the heterologous gene is coupled to the methanol-activated AOX1 promoter [4]; the glucose-depletion activated expression system of *Saccharomyces cerevisiae*[5]; and the arabinose inducible *ara P*_BAD_ promoter in, *E. coli*, [6]. The activation of the *soxS* gene by increasing dissolved oxygen levels could be an additional way for expressing recombinant proteins from *E. coli* without affecting cell growth or metabolism since it does not rely on starvation stress induced by nitrogen, phosphate, or amino acids limitation [7]. Expression of recombinant proteins using high oxygen concentration has attractive properties such as precise control of the induction timing and the elimination of the inducer in the final product or the waste effluents of the bioprocess, making this strategy potentially suitable for production of pharmaceutical-grade proteins [8]. In this report, we describe the expression of recombinant green fluorescent protein from *E. coli* by using the *soxS* promoter and molecular oxygen as an inducer. The expression was compared to other induction procedure and the effect of oxygen on bacterial growth and protein integrity was evaluated.

## Results

### Production of GFP by oxygen-induced promoter in batch growth

**Figure 1:**
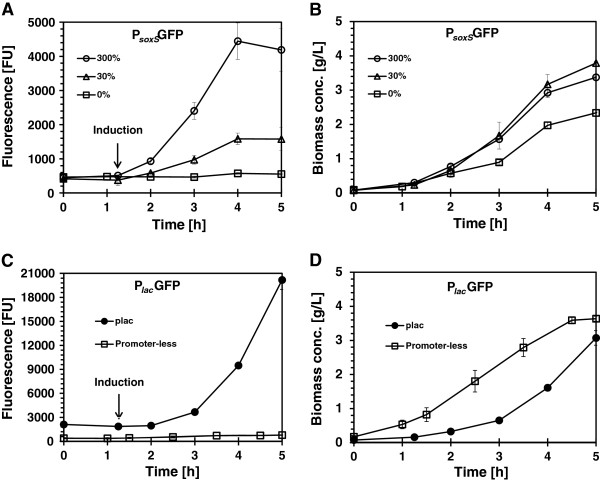
***E. coli *AB1157 growth and GFP production: A, B pAB49 (*soxS *promoter induced by increasing dO**_**2 **_**up to 300%) C, D pAB828 (*lac *promoter) and pAB43 (promoter-less plasmid).** All cultures were controlled at 30% of dO_2_ except those labeled as 300% or 0%. The arrow indicated the time when oxygen or IPTG were added to the culture, the error bars show the standard deviation of experiments performed in triplicate.

The production of recombinant green fluorescent protein (GFP) under the control of the *soxS* (pAB49) and *lac* (pAB828) promoters is shown in Figure 1. *E. coli* AB1157, harboring pAB49, was grown initially at a dissolved oxygen (dO_2_) concentration of 30% air saturation in batch cultures. One hour after inoculation, the dO_2_ concentration was decreased to 0%, maintained at 30% or increased to 300% air saturation and GFP fluorescence was measured (Figure 1A). When the dO_2_ concentration was kept at 0%, GFP fluorescence was 573 FU. At 30% dO_2_ air saturation, GFP fluorescence was 1585 FU, and at an oxygen concentration of 300%, GFP concentration rose to 4500. Thus, the expression level of recombinant GFP increased 7.8-fold with the increased of the dissolved oxygen concentration. No significant changes (p = 0.05) were observed in the specific growth rate of the culture at 30% (1.08/h) and 300% (1.00/h) of dO_2_ (Figure 1B).

**Figure 2:**
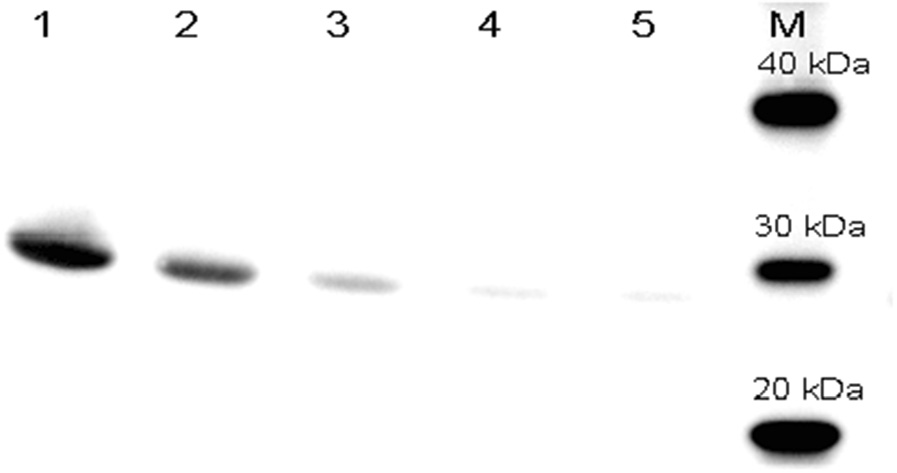
**Western blot of GFP production at 5 h of cultures shown in Figure**1**.** Lane 1, pAB828 induced with 0.5 mM IPTG; lane 2, pAB49 induced by increasing dO_2_ up to 300%; lane 3, pAB49 maintained at 30% dO_2_; lane 4, pAB49 controlled at 0% dO_2_; lane 5, pAB43 negative control with 0.5 mM IPTG. M is molecular weight standard.

GFP expression under the control of the *soxS* promoter was compared to its expression under the IPTG-inducible *lac* promoter. The expression levels from plasmid pAB828 (p_*lac*_-GFP) and plasmid pAB43 (promoter-less GFP) are shown in Figure 1C. The maximum GFP expression by the *lac* promoter, with 0.5 mM IPTG, was 20170 FU at cell concentration of 3.6 g/L (Figure 1C and D). In comparison, the maximum GFP expression in bacteria harboring the promoter-less plasmid (pAB43) was 730 FU which is close to the 573 FU seen with the un-induced (0% dO_2_) culture of cells carrying the p_*soxS*_ plasmid. This construct also showed no response to IPTG as expected (Figure 1C). Western blots of soluble and insoluble fractions were also assayed (data no shown) and GFP was only detected in the soluble fraction of the proteins [9]. To verify that GFP fluorescence intensity was proportional to the amount of GFP produced, western blots were done and results are shown in Figure 2. The highest amounts of GFP were produced by pAB828 induced with IPTG (Figure 2 line 1). For the *soxS* system (pAB49), production of GFP correlated with varying oxygen concentrations (Figure 2 lines 2, 3, and 4). Since the fluorescence of the medium was 380 FU and there was almost no observable GFP signal in line 4 (Figure 2), it can be concluded that the 573 FU produced by soxS-promoter at 0% dO_2_ come from the background effect of the cells and the medium.

The amount of GFP produced was also determined by ELISA; it was calculated that GFP represents 5.1% of total protein produced by cells harboring pAB828 and 1.5% by cells harboring the *soxS* promoter when it was done in batch and 3.4% when it was done in fed batch growth (Table 1). Dividing the fluorescent signal by the amount of GFP produced, specific activities were estimated for both expression systems: 13716 FU/μg of GFP was estimated for pAB828 plasmid and 10736 FU/μg of GFP for the *soxS* promoter plasmid (Table 1).

**Table 1:** **Comparison of specific fluorescence of GFP expressed under control of *lac *** **(pAB828) and *soxS *** **(pAB49) promoters**

**Parameter (units)**	**pAB828 batch, 30% dO**_**2**_	**pAB828 batch, 300% dO**_**2**_	**pAB49 batch, 300% dO**_**2**_	**pAB49 fed-batch, 300% dO**_**2**_
Amount of GFP in the total soluble protein (%)	5.1 ± 0.31	4.9 ± 0.38	1.5 ± 0.03	3.4 ± 0.11
Specific fluorescence (FU/μg GFP)	13753 ± 880	14464 ± 1065	10831 ± 191	11617 ± 1048

± refers to the standard deviation between triplicates.

**Figure 3:**
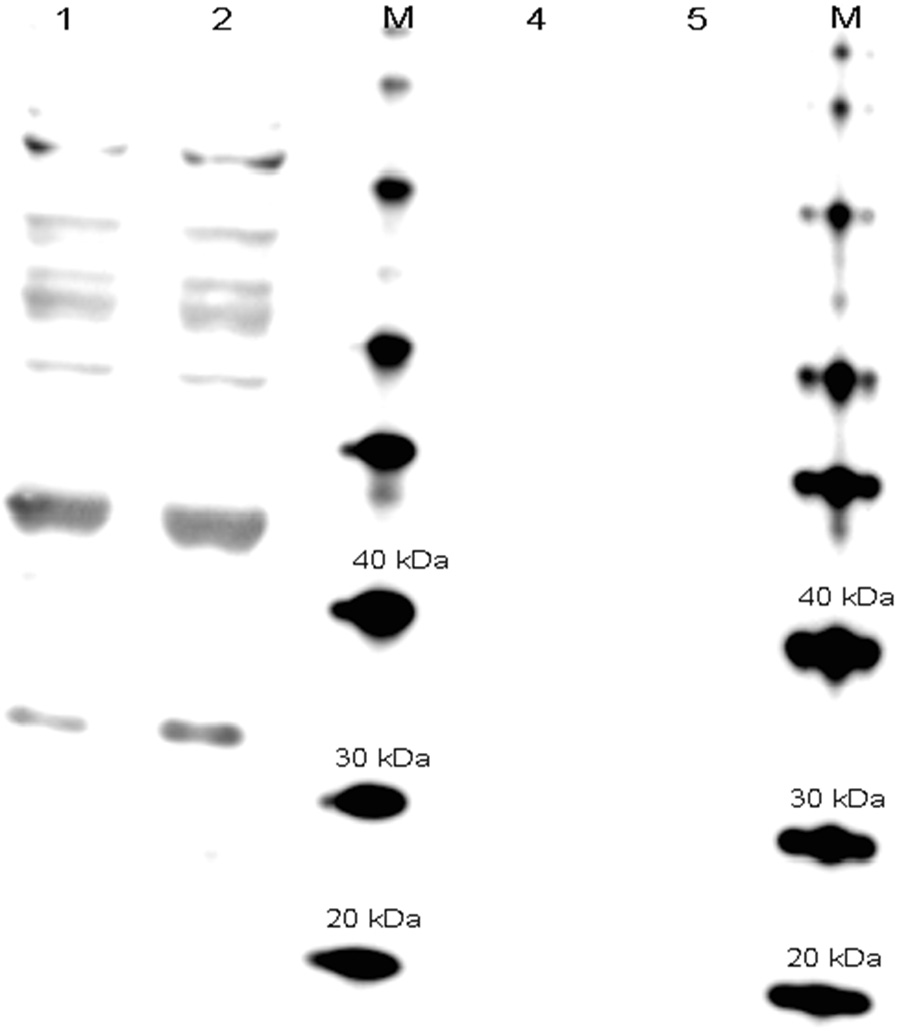
**Oxyblot detection of GFP expression from *E. coli *pAB828 induced with 0.5 mM IPTG at 300% (lane 1) and 30% of dO**_**2 **_**(lane 2).** M refers to molecular weight standards. Lane 4 and 5, negative controls of samples of Lanes 1 and 2. Negative controls were not subjected to the derivatization reaction.

Since high dO_2_ concentrations can increase the intracellular reactive oxygen species [1], the possible effect of the oxygen shift on the integrity of the recombinant protein was evaluated. The protein-carbonyl contents of fresh fermentation samples from pAB828 cultures controlled at 30% and 300% dO_2_ were analyzed using the Oxyblot system to detect protein oxidation (Figure 3). Number of bands showed a pattern of oxidation already appeared at 30% dO_2_. This pattern does not seem to change with higher oxygen concentrations and the lack of oxidized bands at 27 kDa indicates that the recombinant GFP produced at normal or high oxygen concentrations either has no carbonyl groups or they are below detectable levels. The specific fluorescence of the GFP proteins produced at 30% and 300% dO_2_ were similar (Table 1): 13397 FU/μg for the culture exposed to 30% dO_2_ and 14410 for the culture exposed to 300% dO_2_, an indication that the oxygen shift did not affect the integrity of the GFP produced.

### Production of GFP by oxygen-induced promoter in fed-batch growth

**Figure 4:**
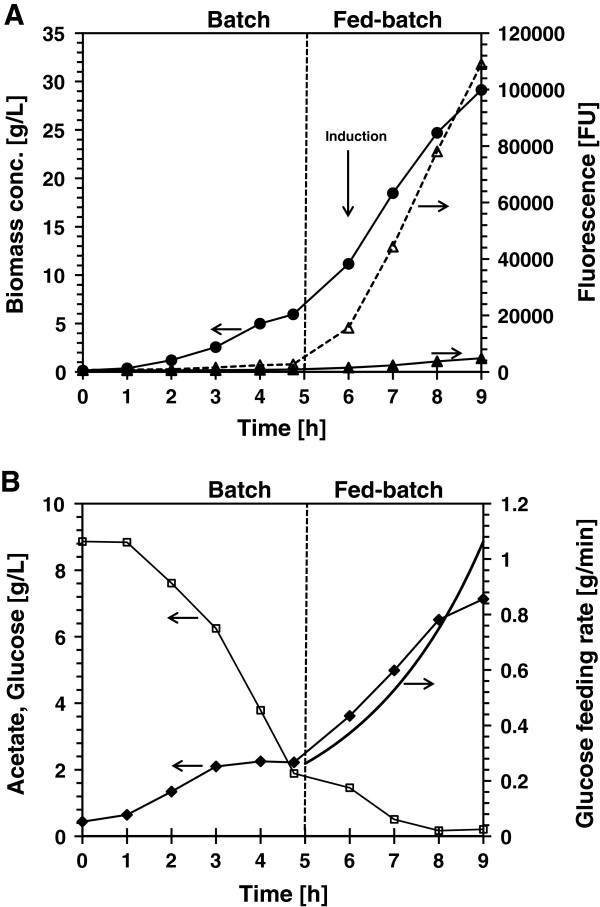
**Production of GFP from the *E. coli *pAB49 (*soxS *promoter) in fed-batch high density growth. A**: Biomass concentration (black circles), total fluorescence (open triangle), and medium fluorescence (black triangle). **B**: Glucose concentration (open square), feeding rate (continuous line), acetate concentration (black diamond).

To test the functionality of the oxygen-induced system, high density fed-batch culture of *E. coli* AB1157 bearing pAB49 was performed by adding glucose exponentially as shown in Figure 4A. After five hours growth, the exponential feeding was initiated to maintain a specific growth rate of 0.35/h. One hour after feeding was initiated; GFP expression was induced by increasing the dO_2_ concentration from 30% to 300% air saturation. After 3 hours at 300% air saturation, GFP fluorescence reached 109000 FU (494 mg/L) representing 3.4% of the total protein and the cell concentration reached 29.1 g/L (DW). The medium fluorescence was only 4.4% of the total signal (Figure 4A). The specific fluorescence reached at 9 h of culture was found to be 11030 FU/μg of GFP produced, similar to the value obtained in the batch growth. The cell growth did not stop after induction and acetate accumulation reached 7.1 g/L (Figure 4B).

## Discussion

The current methods for recombinant protein production in *E. coli* are based on the addition of chemical inducers, temperature shift and nutrient addition or depletion. These methods have their drawbacks: the addition of chemical inducers, such as IPTG can be costly and can contaminate the final product [10,11]; temperature shift can be harmful to the bacteria [12-14]; and nutrient depletion can restrict cell growth or the synthesis of the recombinant proteins [7,14]. As a result, there is an ongoing effort to come up with alternative induction systems that do not affect cell growth or recombinant protein recovery and purification processes [8]. In this work, we have shown that the *E. coli soxS*-promoter expression system can be used to induce recombinant gene expression by increasing the molecular oxygen concentration. The use of molecular oxygen does not negatively affect bacterial growth nor contaminate the recombinant product or the waste effluents of the bioprocess. In addition, this expression system is dynamically responsive, possibly as the result of oxygen diffusion through the cell membrane, allowing full expression to be achieved in less than 90 minutes [15]. The *soxS* induction system was tested by expressing GFP at high cell density culture in a 4 L bioreactor induced by increasing oxygen concentration at OD_600_ of 30. After three hours, the OD_600_ reached 78 and the GFP concentration 0.5 g/L (Figure 4B). The increased oxygen concentration did not affect cell growth properties [1] nor caused the oxidation of the recombinant product as determined by the Oxyblot assay (Figure 3) which is an indicator of protein oxidation [16]. No signal was detected at or below 27 kDa and no difference in the specific fluorescence was observed.

Similar to the oxygen induction approach demonstrated here, heterologous gene expression by thermo-inducible systems is simple and inexpensive. However, the consequences of the heat-shock response as a result of thermo-induction can cause protein denaturation, decrease in specific growth rate, and alteration of central carbon metabolism, presenting a compromise between protein production and stress [12,14]. In comparison with the heat induction method, the increased oxygen strategy did not affect the cell growth nor the quality of the recombinant product. The oxygen induction approach also has an advantage in being tightly controlled and therefore suitable for the expression of potentially toxic products.

Compared with the *lac* promoter-driven expression, the GFP induction by the *soxS* promoter was lower but with similar specific activity. Comparing expression levels is difficult since there are different sequences at the ribosome-binding site (RBS) [15]. It is possible that the expression levels of *soxS* could be increased by using stronger RBS sequences or increasing the strength of the promoter by changing the consensus promoter sequence [17]. Supplying higher concentrations of dissolved oxygen may be difficult and likely expensive when dealing with large bioreactors, but with recent development of oxygen generation technologies [18,19] this method could also be suitable for large scale, however, at present, this technology is limited to applications for laboratory and pilot-scale bioreactors.

## Conclusions

We have demonstrated a novel recombinant protein expression approach based on induction with high oxygen concentration. This method should be considered as an additional way to express recombinant proteins without affecting cell growth and contaminating the pharmaceutical grade products.

## Methods

### Plasmid construction

All plasmid constructs were derived from the pGFPmut3.1 plasmid (Clontech Laboratories, CA) that carries a p_lac_ IPTG-inducible promoter driving the expression of a lacZ leader fused, in frame, to the GFP gene. To make a GFP negative control plasmid (pAB43), the *lac* promoter on the pGFPmut3.1 plasmid was deleted by digestion with SphI and re-ligation overnight. The ligation mix was cut with BamHI to remove any residual parental plasmid prior to transforming the competent cells. Construction of the oxygen-induced plasmid (pAB49) was done in one step: The *lac* promoter of pGFPmut3.1 was replaced with the *soxS* promoter through QuikChange II Site-Directed Mutagenesis (Agilent technologies, CA) with primers *soxS*-GFP-A and *soxS*-GFP-B (Table 2). The mega primer *soxS*-GFP-A contains the native ribosome binding site, *soxS* promoter region and start codon from *E. coli* K-12 genome. Because the pGFPmut3.1 plasmid generates two proteins, a lacZ-GFP fusion protein as well as the GFP protein alone, pAB828 was constructed to delete the lacZ leader but maintain the GFP gene under IPTG control. This was done by site-directed mutagenesis (QuikChange II Site-Kit, Agilent Technologies) using primers GFPmut-A and GFPmut-B (Table 2). All plasmids were confirmed by sequencing.

**Table 2:** Plasmids and oligonucleotides used

**Plasmid, or primer**	**Genotype or sequence**	**Reference or source**
Plasmid		
pAB49	Same as pGFPmut3.1 plus Δplac plus p*soxS*	This study
pAB43	Same as pGFPmut3.1 plus Δplac	This study
pAB828	Same as pGFPmut3.1 plus ::lacZ-GFPmut3.1 fusion	This study
Primers		
GFPmut-A	ATGACCATGATTACGCCAAGCTAGTAGGCCTGATAGTAGACTCTAGAGGATCCCCGGGTA	
GFPmut-B	TACCCGGGGATCCTCTAGAGTCTACTATCAGGCCTACTAGCTTGGCGTAATCATGGTCAT	
*soxS*-GFP-A	TTCTGTGGATAACCGTATTACCGCCTTTGAGTGAGCTGATACCGCTCGCCGCAGCCGAACGACCGAGCGCAGCGAGTCAGTGAGCGAGGAAGCGGAAGTAAATCGCTTTACCTCAAGTTAACTTGAGGAATTATACTCCCCAACAGATGAATTAACGAACTGAACACTGAAAAGAGGCAGATTTATGAGTAAAGGAGAAGAACTTTTCACTGGAGTTGTCCCAATTCTTGTTGAATTAGATGGTGATGTTAATGGGCACAAATTTTCTGTCAGTGGAGAGGGTG	
*soxS*-GFP-B	CACCCTCTCCACTGACAGAAAATTTGTGCCCATTAACATCACCATCTAATTCAACAAGAATTGGGACAACTCCAGTGAAAAGTTCTTCTCCTTTACTCATAAATCTGCCTCTTTTCAGTGTTCAGTTCGTTAATTCATCTGTTGGGGAGTATAATTCCTCAAGTTAACTTGAGGTAAAGCGATTTACTTCCGCTTCCTCGCTCACTGACTCGCTGCGCTCGGTCGTTCGGCTGCGGCGAGCGGTATCAGCTCACTCAAAGGCGGTAATACGGTTATCCACAGAA	

### Bacterial strain, inoculum preparation, and culture media

*Escherichia coli* strain AB1157 (F^-^, thr-l, leuB6, proA2, his-4, thi-1, argE2, lacY1, galK2, rpsL, supE44, ara-14, xyl-15, mtl-1, tsx-33) [20] harboring the pAB49, pAB828, or pAB43 plasmid was grown in batch culture in modified LB medium containing 10 g/L tryptone, 5 g/L yeast extract, 5 g/L NaCl, and 5 g/L K_2_HPO_4_. The pH of the medium was adjusted to 7.0 with 5 M NaOH prior to sterilization. The culture medium was then supplemented with 1 mL/L trace metal solution [21], 5 mM MgSO_4_, 4 g/L of glucose and 100 mg/L of ampicillin. Single colonies of the AB1157 strain transformed with the desired plasmid were grown overnight at 37°C in 100 ml of modified LB containing 100 mg/L of ampicillin. After overnight growth, the culture was inoculated into 5 L bioreactor. The fed-batch medium composition was 10 g/L tryptone, 15 g/L yeast extract, 2.3 g/L KH_2_PO_4_, and 12.5 g/L K_2_HPO_4,_ it was supplement with 2 mL/L trace metal solution, 10 mM MgSO_4_, 7.5 g/L glucose and 100 mg/L of ampicillin. The feeding solution composition was 282 g/L yeast extract, 141 g/L glucose, 100 mg/L ampicillin, and 5 mM MgSO_4_.

### Bioreactor culture conditions

Batch growth was performed in a 5 L B. Braun bioreactor equipped with data acquisition and adaptive dissolved oxygen control system. Temperature was kept at 37°C and pH was maintained at 7.0 with aqueous ammonia (15%, v/v). The bioreactor was inoculated at OD_600_ of 0.2-0.3 and the dissolved oxygen (dO_2_) was measured using polarographic oxygen electrode (Mettler Toledo, Columbus, OH) and controlled at 30% air saturation. Cultures labeled as 0% were performed with limited oxygen concentration (<0.3% air saturation) by bubbling air at 0.075 vvm and agitation of 100 rpm. One hour after inoculation, protein production was induced by the addition of 0.5 mM IPTG to the culture with the *lac* promoter, or by increasing the dO_2_ to 300% air saturation to the culture with the *soxS* promoter [1]. For fed-batch cultures, the growth started at batch mode with initial volume of 4 L, followed by predetermined exponential feeding algorithm according to the following equation [22]:

$$F\left(t\right)=\left(\frac{\mu_{\mathrm{set}}}{Y_{x/s}}+m\right)*\frac{X_{F}*V_{F}}{S_{F}}*e^{\mu_{\mathrm{set}}\left(t‒t_{F}\right)}$$

F(t) = feed rate (L/h), μ_set_ (0.35 1/h) = specific growth rate, Yx/s (1.28 g/g) = biomass yield on glucose estimated from exponential growth phase in batch cultures, m (0.12 g/g h) = maintenance coefficient, the V_F_ and X_F_ are the volume and biomass concentration in the bioreactor at the onset of fed-batch procedure, S_F_ (141 g/L) = glucose concentration in the feed solution. Samples were collected at regular intervals to determine cell growth, protein production and metabolite concentrations.

### Analytical methods

Cell growth was followed by measuring the OD at 600 nm (Ultrospec 3000 UV/Visible spectrophotometer, Pharmacia Biotec); measurements were converted to dry cell weight by using a calibration curve of dried samples. Glucose concentration was determined by YSI 2700 Biochemistry Analyzer (YSI Instruments, Yellow Springs, OH), fluorescence measurement was performed on the fluorometric plate reader SpectraMax Gemini XS (Molecular Devices) with a 485/515 nm filter set and 515 nm cutoff. Fluorescence intensity from fresh fermentation samples diluted 1:10 in water was analyzed in triplicate in a 96-well plate. Acetate concentration was measured by high-performance liquid chromatography (Hewlett Packard/Agilent 1100 Series, Santa Clara, CA) with an Aminex HPX-87H column (Bio-Rad Laboratories, Hercules, CA) equipped with a photodiode array detector. A mobile phase of 5 mM H_2_SO_4_ was used at 0.6 ml/min, run at 35°C.

### Western blot analysis and ELISA

Cell pellets from fermentation samples were washed twice in 50 mM sodium phosphate buffer pH 7.4, re-suspended in 300 μl of the same buffer, and disrupted by sonication in a series of 4 × 15 seconds in a cold bath. Protein concentration was determined by the Bradford method. Samples containing 6 μg protein were separated by SDS-PAGE on NuPAGE® Novex® 4-12% Bis-Tris Gel (Life Technologies, Grand Island, NY) and transferred onto nitrocellulose membranes using the iBlot® Gel Transfer Stacks (Life Technologies, Grand Island, NY), according to manufacturer’s instructions. Membranes were then incubated for 30 min in blocking buffer (WesternBreeze® Blocker/Diluent part A and B, Life Technologies) on a rocker at room temperature. Primary mouse monoclonal anti-GFP antibody (Sigma-Aldrich, St. Louis, MO) was added for 1 h (diluted 1:5,000) followed by goat anti-mouse secondary antibody (KPL, Gaithersburg, MD) at 1:10,000. Detection was done using the SuperSignal West Pico Chemiluminescent Substrate detection kit (Thermo Scientific, Rockford, IL). Same cell-free extract supernatants were analyzed using a GFP ELISA kit (cat. no. AKR121; Cell Biolabs, San Diego, CA).

### Determination of oxidative stress on recombinant product (protein-carbonyl content)

The presence of carbonyl groups in the protein side chains of crude protein extracts was determined by gel electrophoresis and Western blotting using Millipore’s OxyBlot kit (Catalog No. S7150) [23]. Cell pellets from fresh fermentation samples of pAB828 cultures controlled at 30% or 300% dO_2_ were washed and disrupted by sonication in the presence of 50 mM of DL-Dithiothreitol as described above. After determining protein concentrations, 8 μg of total protein were used for derivatization with 2,4-dinitrophenyl hydrazine (DNPH) following manufacturer’s instruction, and a parallel sample was used as negative control by substituting the derivatization-control solution for the DNPH Solution. Derivatized samples and negative controls were separated by SDS-PAGE and transferred onto nitrocellulose membranes as previously described above. Membranes were incubated for 1 h in blocking/dilution buffer (1% BSA/PBS-T) on a rocker at room temperature, followed by 1 h in the primary antibody solution (Rabbit Anti-DNP diluted 1:150). After two rinses with 1X PBS-T, the membrane was incubated for 1 h in the secondary antibody solution (Goat Anti-Rabbit IgG HRP-conjugated diluted 1:300). The SuperSignal West Pico Chemiluminescent Substrate detection kit (Thermo Scientific, Rockford, IL) was used for signal development.

## Abbreviations

GFP: Green fluorescent protein; soxS: Transcriptional dual regulator of superoxide response regulon; FU: Fluorescence units; DW: Dry weight; dO2: Dissolved oxygen; IPTG: Isopropyl β-D-1-thiogalactopyranoside; ELISA: Enzyme-linked immunosorbent assay.

## Competing interests

The authors declare that they have no competing interests.

## Authors’ contributions

AB designed and conducted the experiments. NM helped with molecular cloning and data interpretation. AB and JS together analyzed the data and wrote the manuscript. All the authors read and approved the final manuscript.
